# Geometric and density relationships of calcification clusters in carotid atherosclerosis

**DOI:** 10.1177/19714009261450380

**Published:** 2026-05-04

**Authors:** Juul Bierens, Roberta Scicolone, Valentina Nardi, John Benson, Antonella Balestrieri, Antonio Incollu, Jasjit S. Suri, Jae Song, Giuseppe Lanzino, Marianne Eline Kooi, Luca Saba

**Affiliations:** 1 Department of Radiology and Nuclear Medicine, 199236 Maastricht University Medical Center, Maastricht, The Netherlands; 2 CARIM Cardiovascular Research Institute Maastricht, Maastricht University, Maastricht, The Netherlands; 3 Department of Radiology, 3111 University of Cagliari, Cagliari, Italy; 4 Department of Cardiovascular Diseases, 6572 Mayo Clinic, Rochester, MN, USA; 5 Department of Radiology, 6572 Mayo Clinic, Rochester, MN, USA; 6 Stroke Monitoring and Diagnostic Division, AtheroPoint, Roseville, CA, USA; 7 Department of Radiology, 6572 University of Pennsylvania, Philadelphia, PA, USA; 8 Department of Neurosurgery, 6572 Mayo Clinic, Rochester, MN, USA

**Keywords:** carotid plaque, calcification, geometry, cluster analysis, CT, surrogate biomechanics

## Abstract

**Background:**

Carotid artery calcification represents a common feature of atherosclerotic plaques. However, the geometric relationships of calcific clusters have not been systematically investigated. This study aimed to develop a geometry-based atlas, independent of symptom status, characterizing the three-dimensional properties of calcium clusters within carotid plaques and quantifying population-level distributions and data-driven couplings between size, shape, and density.

**Methods:**

Standardized three-dimensional calcium masks of clinical CT scans were analyzed using connected-component labeling. Extracted features included cluster volume, aspect ratio, eccentricity, compactness, and CT attenuation values (mean μ, standard deviation σ). Associations between cluster features were assessed using Spearman correlations with Benjamini–Hochberg false discovery rate correction. Independent associations were determined using partial Spearman correlation and restricted cubic spline regression.

**Results:**

Among 107 plaques, 149 distinct clusters were identified, most frequently presenting as a single elongated cluster (aspect ratio = 2.23). Independent associations (all *p* < 0.001) were identified for: eccentricity with μ (ρ = −0.39), volume with μ (ρ = 0.47), compactness with μ (ρ = 0.39), and volume with eccentricity (ρ = 0.82). Multi-cluster plaques exhibited smaller mean cluster volume (76.1 vs 359.5 mm^3^; *p* < 0.001) and lower eccentricity (0.5 vs 1.6; *p* < 0.001) compared with single-cluster plaques.

**Conclusion:**

Carotid calcification most frequently manifests as a single elongated cluster, with robust couplings between cluster size, shape, and density. These geometric archetypes provide a quantifiable framework for future biomechanical and biological studies of plaque vulnerability.

## Introduction

Calcification is a hallmark of atherosclerotic plaque, arising from active remodeling of the arterial wall driven by inflammation, extracellular matrix turnover, and osteogenic trans-differentiation of vascular smooth muscle cells. Mineralization progresses through extracellular vesicles, apoptotic bodies, and matrix-mediated nucleation.^1^ In the carotid arteries, contemporary imaging is shifting attention from stenosis measurements alone toward a more refined risk assessment including plaque morphology and composition, to improve risk stratification and clinical decision-making.^2–5^ Yet, carotid calcification is often excluded from such frameworks, as evidence linking carotid calcification to cerebrovascular risk is inconsistent and its prognostic significance remains uncertain.^2,6^

A recent meta-analysis reported no overall association between the presence or volume of calcification and plaque rupture but identified a reduced rupture risk associated with calcification in plaques with ≥50% stenosis and an increased rupture risk associated with calcification in non-stenotic plaques.^6^ Such discrepancies likely reflect the heterogeneity of calcification phenotypes.^7^ Rather than a uniform entity, calcification encompasses a spectrum of morphologies and spatial patterns that may correspond to distinct stages of atherosclerosis and confer differential mechanical stability. From a mechano-biological perspective, microcalcifications embedded within a thin fibrous cap can amplify local stress concentrations and promote rupture, whereas confluent macrocalcifications are often associated with fibrotic stabilization and a reduced risk of rupture.^7,8^ Thus, the size, shape, and spatial clustering of mineral deposits influence stress fields and the likelihood of cap rupture.^9–14^

Despite these insights, carotid-specific data on the three-dimensional geometric organization of calcification, how clusters are sized, shaped, distributed, and positioned, remains limited and heterogeneous across cohorts and imaging protocols.^3,15–20^ No systematic morphometric framework exists from which reproducible calcification phenotypes can be derived. This study therefore aimed to provide a comprehensive characterization of carotid calcific clusters, quantifying their morphometric characteristics and their relation to CT attenuation. This work is intended as a methodological characterization framework. Clinical validation linking these morphometric to cerebrovascular risk factors and patient outcomes is planned as a subsequent step.

## Materials and methods

### Study Design and cohort

A cross-sectional analysis of three-dimensional carotid calcium masks derived from isotropically reconstructed clinical CTA scans was performed. This study included consecutive adult patients (≥18 years) who belonged to one of two clinical scenarios: (i) symptomatic patients evaluated in the acute phase, in whom CTA was performed as part of standard clinical care or (ii) asymptomatic patients referred for CTA after duplex ultrasound had identified either a carotid stenosis greater than 50% and/or ultrasound features suggestive of plaque vulnerability. Non-adult patients and any subjects with a contraindication to iodinated contrast media according to routine clinical practice (e.g., prior severe contrast reaction or other standard contraindications) were excluded. The study was deliberately symptom-agnostic: no clinical history, signs/symptoms, or outcomes were considered. All segmentation parameters were kept constant across the cohort, in accordance with consensus recommendations for standardizing carotid imaging.^3,21^

Ethical approval was obtained from the Institutional Review Board of the University of Cagliari (Comitato Etico Indipendente; PG/2019/13156), which waived informed consent, and the study complied with the Declaration of Helsinki.

### CTA acquisition

CTA of the carotid arteries was performed using a standardized protocol (120 kVp; 280–320 mAs) on multi-detector CT scanners (Brilliance, Philips Healthcare, the Netherlands; Aquilion, Canon Medical Systems Corporation, Japan; Revolution, GE Healthcare, USA). Examinations extended from the aortic arch to the carotid siphon in caudo-cranial direction and were acquired before and after contrast administration. Angiographic phase was obtained with 50–70 ml of pre-warmed contrast (Ultravist 370) injected at 4 ml/s, with bolus tracking (threshold +80 HU) to optimize timing. When the threshold was reached, scanning started after a 4-second delay. Technical parameters included 0.6 mm slice thickness, 0.3 mm interval, 512 × 512 matrix, 14–19 cm field of view, and C-filter reconstruction.

### Segmentation and labeling

Calcium at the carotid bifurcation was identified using a validated CTA pipeline comprising: (i) intensity pre-processing and normalization; (ii) intensity-guided (≥130 HU) segmentation with anatomical constraints; (iii) morphological cleaning; (iv) 3D connected-component labeling; and (v) visual quality control to exclude adjacent bony artifacts. This pipeline was entirely developed using Python, integrating custom-built scripts for segmentation and component labeling. The methodology is consistent with literature advocating reproducible approaches and transparent reporting of parameters.^3^

### Feature extraction

A calcification cluster was defined as a single discrete calcification. For each cluster, the following features were calculated: volume (mm^3^); maximum and minimum linear dimensions (mm) from the minimum oriented bounding box; aspect ratio (ratio of maximum and minimum dimension); eccentricity (elongation); compactness (sphericity); CT mean attenuation (μ HU) and attenuation heterogeneity (standard deviation of the attenuation, σ HU); and centroid coordinates (x, y, z, mm). At the patient/plaque level, number of clusters and computed aggregated summaries were recorded.

### Statistical analysis

Given the non-Gaussian distributions of variables, variables were summarized as medians with interquartile ranges (IQR). Associations between the various per-cluster features were assessed using Spearman’s rank correlation coefficient (ρ). To control for multiple testing, *q*-values were calculated with the Benjamini–Hochberg false discovery rate (FDR) method (significance threshold *q* < 0.05).

To evaluate independent associations, partial Spearman correlation analyses were performed, quantifying correlations between two variables while adjusting for the other per-cluster features. To further characterize relationships, multivariable linear regression models incorporating all cluster variables were fitted using restricted cubic splines (RCS; 3 knots) and visualized. Non-linearity was assessed using Wald χ^2^ tests from regression ANOVA. Robustness was enhanced by identifying influential observations with multiple influential diagnostics (Cook’s distance, leverage, DFBETA); data points flagged by any of these criteria were excluded. 95% confidence intervals for Spearman and partial Spearman correlations were calculated using a bootstrap percentile method with 1000 resamples.

To examine relationships between per-plaque cluster counts and per-cluster features, the primary analysis dichotomized plaques into single-versus multi-cluster calcifications, given the limited number of plaques with >1 cluster. Differences were assessed using the Mann–Whitney U test. As a sensitivity analysis, plaques were additionally stratified by exact cluster count and compared using the Kruskal–Wallis test, with significant findings further examined by pairwise Mann–Whitney U tests with Benjamini–Hochberg adjustments. All analyses were performed in Python (SciPy/NumPy/Pandas) and R.

## Results

### Cluster characteristics

A total of 149 calcification clusters were identified across 107 plaques, derived from 61 patients (mean age: 70 ± 9 years; 54% male; Table 1). The distribution of calcification clusters per plaque is also detailed in Table 1, with single-cluster plaques representing the predominant pattern (81%). Comprehensive descriptive statistics are provided in the table. In brief, single clusters demonstrated a median volume of 172.5 mm^3^ [43.3–418.4], a maximum dimension of 6.2 mm [4.3–11.3], and a minimum dimension of 3.8 mm [1.2–4.9]. Shape descriptors included a median aspect ratio of 2.23 [1.41–3.03], compactness of 2.87 [1.51–4.31], and eccentricity of 1.16 [0.40–2.18]. Median attenuation of clusters was 405 HU [267–566] with a standard deviation of 250 HU [125–350].

**Table 1.** Baseline characteristics of patients, plaques, and calcification clusters.

Patient characteristics (n = 61)	
Age, years	70 ± 9
Male sex	33 (54)
Symptomatic	31 (51)
Per-plaque features (*n* = 107)	
Total calcified volume, mm^3^	323.7 [152.0–544.1]
Fragmented calcification (>1 cluster)	20 (19)
Degree of stenosis, %	51 ± 22
Morphometric per-cluster calcification features (*n* = 149)	
Volume, mm^3^	172.5 [43.3–418.4]
Maximum dimension, mm	6.2 [4.3–11.3]
Minimum dimension, mm	3.8 [1.2–4.9]
Aspect ratio	2.23 [1.41–3.03]
Compactness	2.87 [1.51–4.31]
Eccentricity	1.16 [0.40–2.18]
Density per-cluster calcification features (*n* = 149)	
μ attenuation, HU	405 [267–566]
σ attenuation, HU	250 [125–350]

Values are mean ± SD, *n* (%), or median [IQR].

### Relationship between calcification size, shape, and density

**Figure 1.**
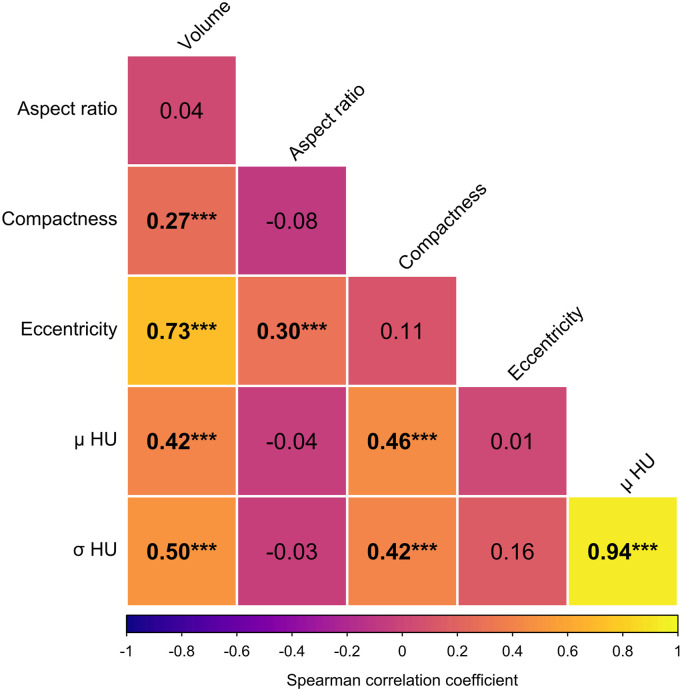
Spearman correlation matrix of per-cluster features of calcification clusters. Correlations that reached statistical significance are highlighted in bold. *** indicates *p* < 0.001.

Figure 1 depicts unadjusted Spearman correlation coefficients for per-cluster calcification features. Cluster volume was significantly correlated with compactness (ρ = 0.27 [95% CI: 0.09–0.43]; q < 0.001), eccentricity (ρ = 0.73 [95% CI: 0.63–0.81]; q < 0.001), mean attenuation (ρ = 0.42 [95% CI: 0.26–0.56]; q < 0.001), and attenuation heterogeneity (ρ = 0.50 [95% CI: 0.36–0.62]; q < 0.001). Mean attenuation was strongly correlated with σ attenuation (ρ = 0.94 [95% CI: 0.90–0.96]; q < 0.001) and moderately with compactness (ρ = 0.46 [95% CI: 0.28–0.61]; q < 0.001). Compactness and σ attenuation were also correlated (ρ = 0.42 [95% CI: 0.25–0.56]; q < 0.001). Aspect ratio demonstrated a modest correlation with eccentricity (ρ = 0.30 [95% CI: 0.07–0.52]; q < 0.001).

**Figure 2.**
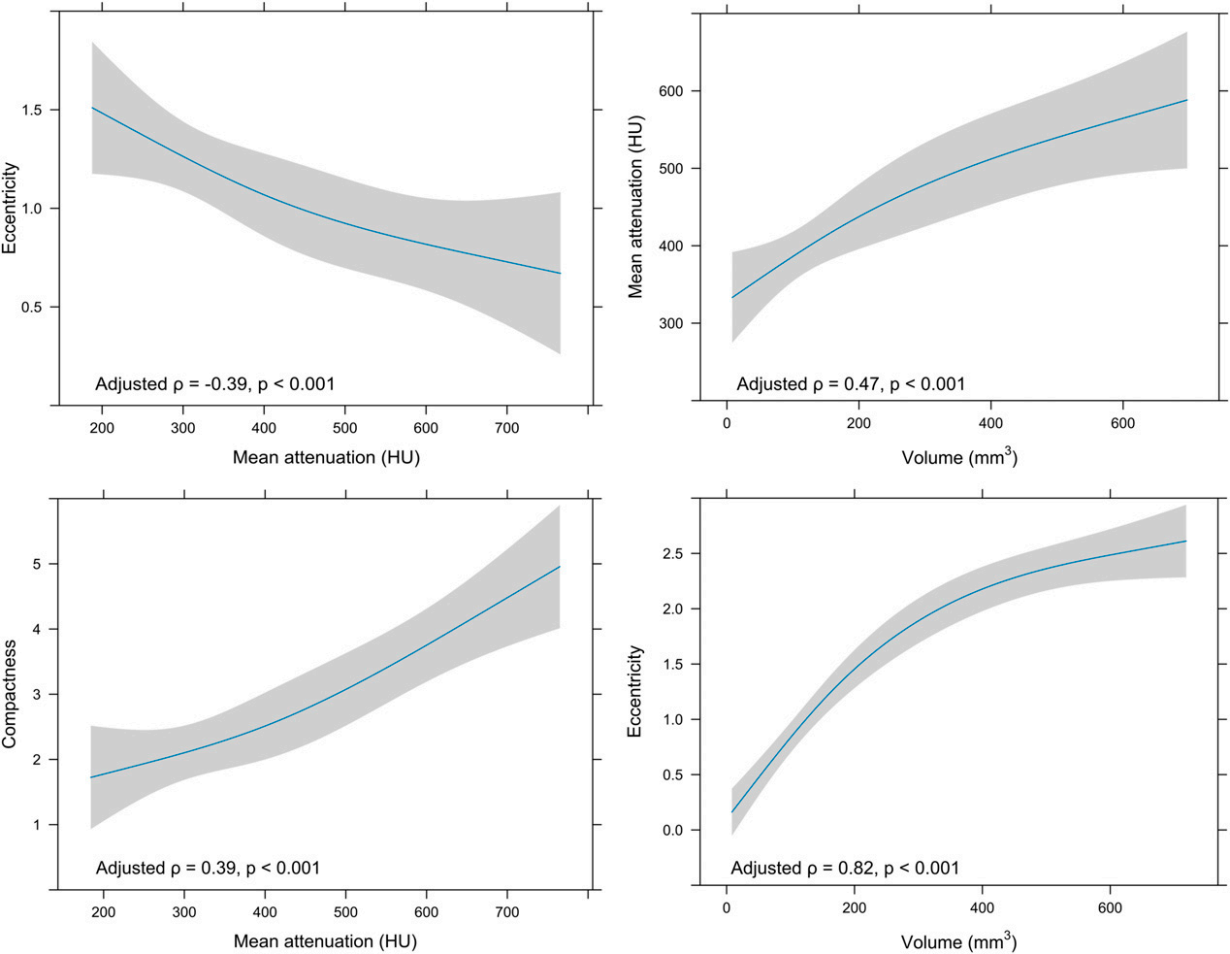
Adjusted restricted cubic spline curves (three knots) with corresponding adjusted Spearman correlations for associations identified as significant in unadjusted analyses. Only the correlation between volume and eccentricity is non-linear.

In a subsequent analysis, robust independent effects were evaluated while controlling for influential datapoints. Figure 2 presents RCS plots of significant partial Spearman correlations. The associations of volume with eccentricity (ρ = 0.82 [95% CI: 0.76–0.87], p < 0.001), volume with mean attenuation (ρ = 0.47 [95% CI: 0.29–0.61], p < 0.001), and mean attenuation with compactness (ρ = 0.39 [95% CI: 0.23–0.54], p < 0.001) remained significant. Additionally, an independent moderate negative correlation between calcification attenuation and eccentricity emerged (ρ = −0.39 [95% CI: −0.53–−0.23], *p* < 0.001), which was obscured in the unadjusted analysis due to positive confounding with volume. Wald χ^2^ testing demonstrated a significant non-linear association between cluster volume and eccentricity (*p* < 0.001), whereas all other evaluated relationships were consistent with linearity (*p* > 0.05).

### Relationship between cluster fragmentation and size, shape, and density

**Figure 3.**
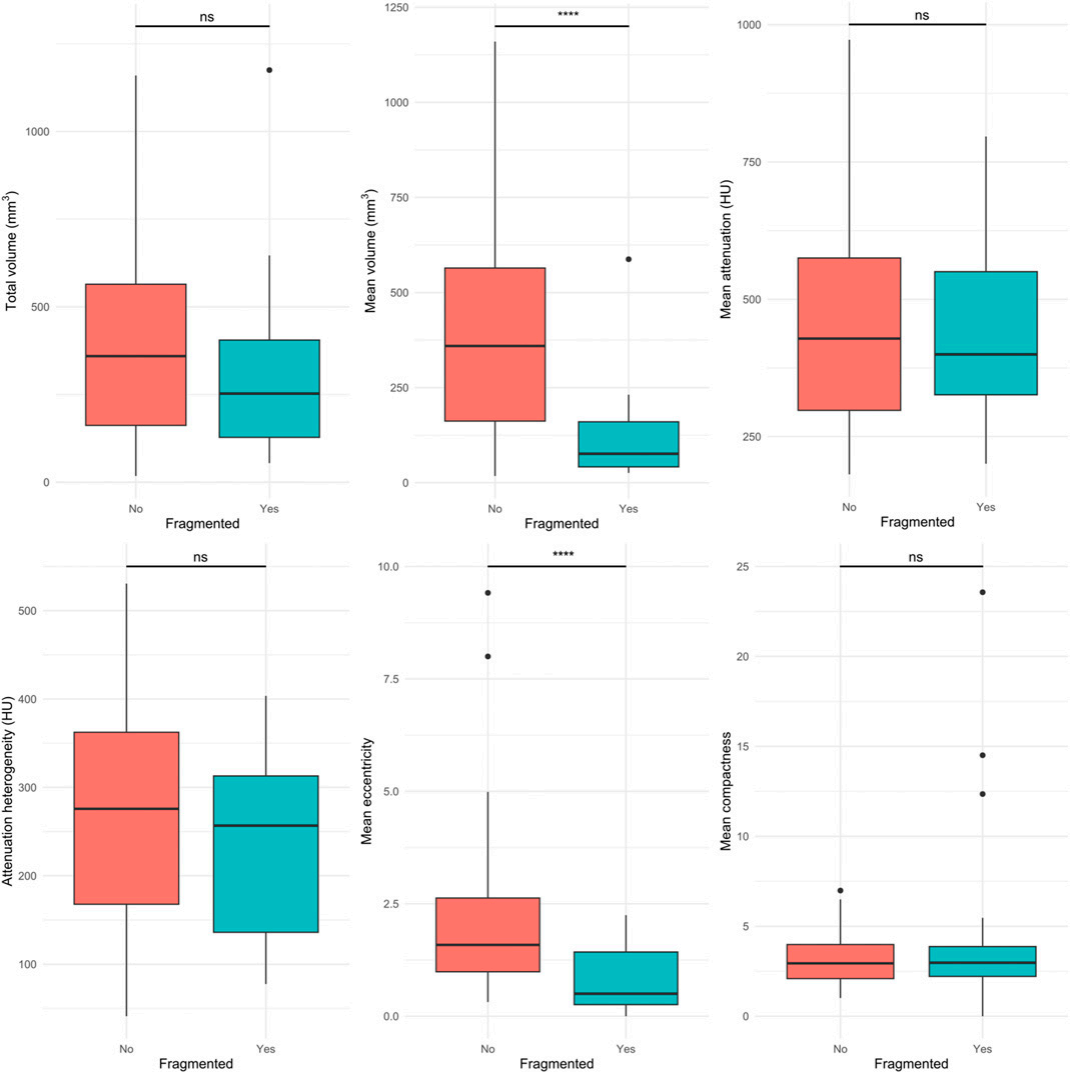
Boxplots of per-cluster characteristics comparing continuous single-cluster versus fragmented multi-cluster calcifications. **** indicates *p* < 0.0001.

**Figure 4.**
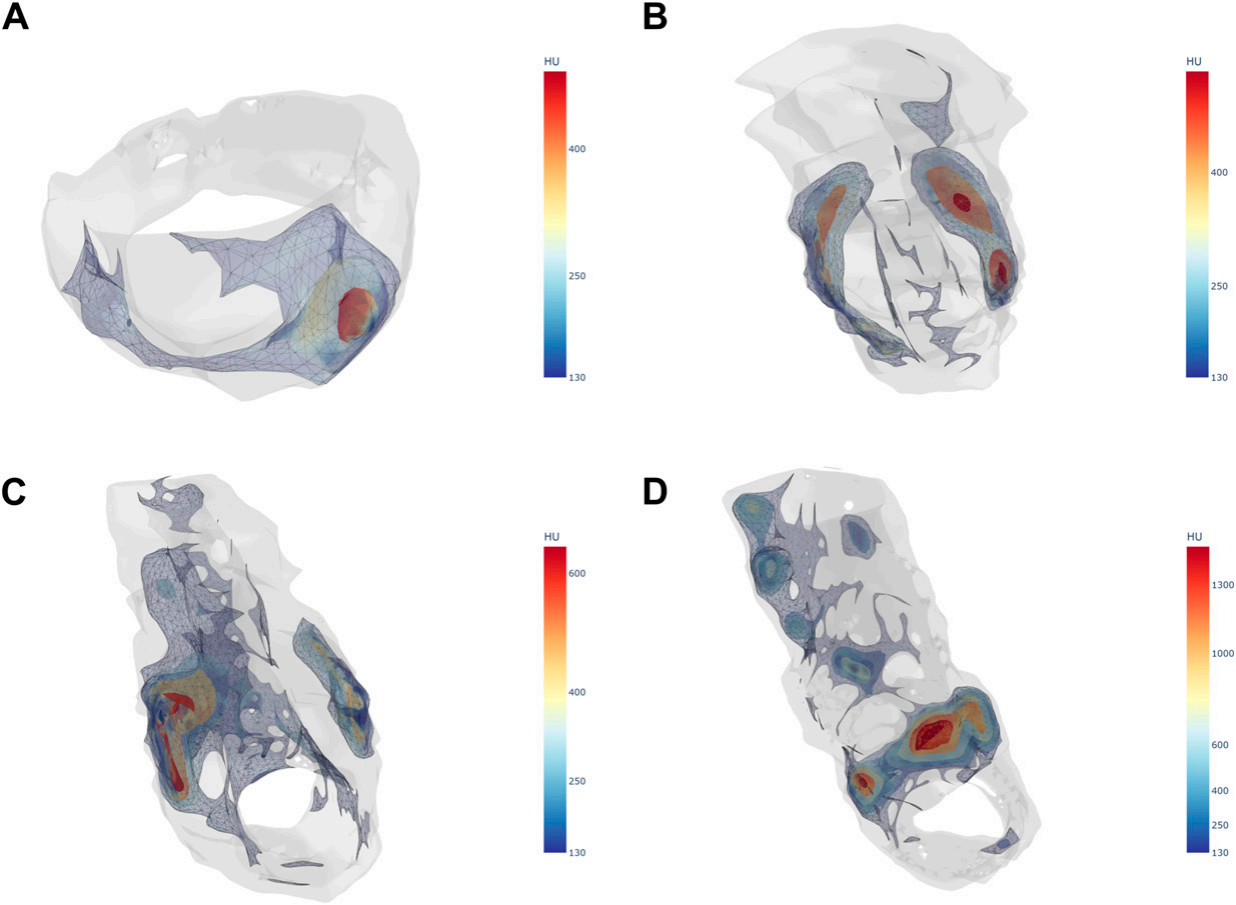
Representative images of the cluster analysis for single- (a) and multi-cluster (b-d) calcifications.

Figure 3 presents boxplots of per-cluster characteristics comparing continuous single-cluster with fragmented multi-cluster calcifications. Mann–Whitney U tests demonstrated that multi-cluster plaques exhibited significantly lower mean cluster volume (76.1 [95% CI: 41.2–161.2] vs 359.5 [95% CI: 160.6–570.8] mm^3^; *p* < 0.001) and eccentricity (0.5 [95% CI: 0.3–1.4] vs 1.6 [95% CI: 1.0–2.6]; *p* < 0.001) compared with single-cluster plaques. No significant differences were observed for total cluster volume, attenuation, attenuation heterogeneity, and compactness. Figure 4 provides representative examples of single- and multi-cluster calcifications.

These findings were confirmed in the sensitivity analysis stratified by exact cluster count per plaque. Kruskal–Wallis tests revealed significant associations of cluster count with mean cluster volume (*p* < 0.001) and eccentricity (*p* < 0.001), whereas total cluster volume, attenuation, attenuation heterogeneity, and compactness remained non-significant. Post hoc pairwise Mann–Whitney U tests identified significant differences between plaques with one versus two clusters for mean cluster volume (q = 0.046) and eccentricity (q = 0.025).

## Discussion

Carotid atherosclerotic calcification most frequently occurs as a single cluster with elongated morphology (aspect ratio ≈ 2), moderate irregularity, and intermediate attenuation. Strong dependencies were observed between calcification size, shape, and density and identified that multi-cluster calcifications represent a distinct morphological phenotype compared to single-cluster calcification.

Larger calcifications exhibit both higher mean attenuation and greater attenuation heterogeneity. This association has been demonstrated before and is biologically consistent with the process of mineralization, as calcification growth involves accumulation of hydroxyapatite crystals and gradual consolidation, resulting in reduced porosity and increased mineral concentration, which manifests as increased radiodensity.^22,23^ However, this process is not spatially uniform: cores become progressively more compact and mineralized, whereas peripheral regions, representing more recent deposition, remain less dense.^24^ The resulting increasing gradient between core and periphery provides an explanation for the increased attenuation heterogeneity observed in larger, denser calcifications.

Calcification volume was also strongly correlated with eccentricity, but not with aspect ratio. Although both parameters describe morphological irregularity, their definitions capture distinct features: eccentricity reflects deviation from sphericity, incorporating both overall geometry and local surface asymmetries, whereas aspect ratio only reflects overall geometry. These findings suggest that calcification growth promotes more irregular and asymmetric geometry, while overall elongation remains relatively stable. This is consistent with previous morphometric work, where one study reported increased eccentricity in larger calcifications, while another found no change in aspect ratio.^25,26^ After multivariable adjustments, increased density was independently associated with reduced eccentricity, while aspect ratio remained unchanged. Increased density was also consistently related to increased compactness. These results suggest that dense calcifications adopt a rounder, more uniform morphology without altering overall dimensions. Biologically, this may reflect the fact that denser mineralized regions are more mechanically rigid and resistant to deformation, thereby suppressing the development of asymmetric growth.^7^ Notable, the inverse relationship between density and eccentricity was only apparent in multivariate analysis. In unadjusted analysis, the positive correlation of volume with both density and eccentricity obscured the independent negative effect. Once volume was accounted for, the intrinsic relationship became evident.

The per-plaque analysis demonstrated that multi-cluster, fragmented calcifications influenced cluster eccentricity and volume, without altering total calcified volume, density or aspect ratio. This observation aligns with the concept that single-cluster calcifications represent a more advanced stage of growth, arising from the coalescence of smaller clusters.^24^ As two discrete calcifications expand and merge, their combined volume increases to approximately the sum of the original clusters, thereby doubling the mean cluster volume, while the overall calcified volume remains unchanged. The smaller, isolated clusters exhibit a single dense core, reflected as a spherical shape with low eccentricity. In contrast, the fusion of adjacent calcifications yields a structure in which the dense cores are displaced from the new geometric center, producing a more heterogenous distribution and, consequently, higher eccentricity.^24^

Taken together, these findings suggest that advanced descriptors of calcification shape and density, such as eccentricity and compactness, may be more sensitive descriptors of biologically and mechanically relevant changes in atherosclerotic calcifications than aspect ratio or size. Unlike aspect ratio, eccentricity captures internal asymmetries and protrusions that may reflect underlying growth dynamics or biomechanical stress, features that are likely to influence plaque stability. Previous analyses of calcification morphology have shown that more or smaller calcifications are associated with plaque vulnerability and clinical symptoms.^8,27^ Future studies linking detailed calcium geometry and density with markers of vulnerability or clinical outcomes will be essential to determine whether cluster phenotypes can provide more sensitive markers of calcification biology and plaque destabilization. The present study should therefore be understood as a methodological foundation: a systematic characterization of calcification morphology and density using a standardized three-dimensional pipeline, which provides the descriptives necessary for subsequent clinical validation studies examining associations with cardiovascular risk factors and cerebrovascular events.

Beyond their descriptive value, these morphometric and densitometric parameters have further potential for translational research. Incorporating detailed descriptors of calcification phenotype into biomechanical models may improve estimation of local stress amplification to enhance differentiation between calcification morphologies linked to stability or rupture. Combined imaging with^18^ F-NaF PET, which detects active mineralization, could further integrate structural and metabolic information, providing a more comprehensive understanding of plaque biology.^28–30^ In parallel, imaging-based reporting frameworks, such as Plaque-RADS, could be expanded to include calcific architecture as a formal modifier, alongside established markers like intraplaque hemorrhage and surface irregularity, further moving towards a comprehensive assessment of plaque vulnerability.^2,21^

A major strength of the work lies in the use of a consistent three-dimensional processing pipeline, ensuring that all plaques were analyzed with identical segmentation parameters and feature extraction methods. Additionally, the spatial distribution of calcific clusters was represented through three-dimensional centroid mapping, providing a detailed visualization of plaque architecture. The application of FDR corrections, multivariable RCS, and identification and exclusion of influential outliers strengthened the statistical rigor of the analysis, enabling the detection of robust, independent associations among morphometric and densitometric calcification parameters.

Several limitations should be acknowledged. First, the cohort was recruited from a single center using a standardized acquisition protocol, which ensures internal consistency but limits generalizability. Second, no formal cross-scanner adjustment or calibration was performed across CT scanners. While scanners employed identical tube voltage and reconstruction settings, residual inter-scanner variability may have introduced subtle differences in image texture and attenuation characteristics that could influence morphometric feature extraction. Third, lumen–wall regions of interest (ROIs) were not available for radial and circumferential normalization of cluster positions and prevented topographic standardization of centroid locations. Fourth, the segmentation process may be sensitive to spatial resolution limits, voxel anisotropy, and partial-volume effects, which can influence the accuracy of calcium delineation and feature measurement. These factors remain an inherent technical limitation of clinical CTA datasets.^3,21^ Finally, as calcification phenotypes were derived exclusively from CTA data, biological interpretation of morphometric parameters remains exploratory.

In conclusion, carotid artery calcification most frequently presents as a single, elongated cluster with moderate irregularity. The observed relationships among size, shape, and density reflect a structural organization that may influence the distribution of mechanical stresses within the plaque. Variations in mineral density within individual clusters likely arise from differences in crystal composition, packing density, porosity, or the presence of multiple mineral phases. Importantly, such heterogeneity has direct mechano-biological implications, as materials with non-uniform density can respond unevenly to mechanical loading, potentially affecting plaque stability and the risk of rupture.
